# Definition of a Dietary Pattern Expressing the Intake of Vegetables and Fruits and Its Association with Intestinal Microbiota

**DOI:** 10.3390/nu15092104

**Published:** 2023-04-27

**Authors:** Toshitaka Yamauchi, Naoko Koyama, Ayumi Hirai, Hiroyuki Suganuma, Shigenori Suzuki, Koichi Murashita, Tatsuya Mikami, Yoshinori Tamada, Noriaki Sato, Seiya Imoto, Ken Itoh, Shigeyuki Nakaji

**Affiliations:** 1 Innovation Division, KAGOME CO., LTD., 17 Nishitomiyama, Nasushiobara 329-2762, Japan; 2 Innovation Center for Health Promotion, Hirosaki University Graduate School of Medicine, 5 Zaifu-cho, Hirosaki 036-8562, Japan; 3 Human Genome Center, The Institute of Medical Science, The University of Tokyo, Shirokanedai, Minato-ku, Tokyo 108-8639, Japan; 4 Department of Vegetable Life Science, Hirosaki University Graduate School of Medicine, Hirosaki 036-8562, Japan; 5 Department of Stress Response Science, Center for Advanced Medical Research, Hirosaki University Graduate School of Medicine, 5 Zaifu-cho, Hirosaki 036-8562, Japan; 6 Department of Social Medicine, Hirosaki University Graduate School of Medicine, 5 Zaifu-cho, Hirosaki 036-8562, Japan

**Keywords:** microbiota, vegetables and fruits, dietary patterns, nutrients, diversity

## Abstract

Daily dietary habits directly or indirectly influence the intestinal microbiota, and the resulting changes in its composition and metabolic activity alter the health conditions of the host. Although many studies have analyzed the association between individual nutrients/food items and intestinal microbiota, the assessment of the diet and intestinal microbiota from a macroscopic perspective has not yet been performed in Japan. Therefore, we focused on vegetables and fruits and aimed to identify dietary patterns of high intake of these foods and to examine their relationship with the intestinal microbiota. This cross-sectional study included 1019 healthy individuals aged ≥20 years in a rural area in northern Japan. Six dietary patterns were detected by factor analysis using the brief-type self-administered diet history questionnaire (BDHQ) data to identify the “vege pattern”, which was the dietary pattern rich in vegetables and fruits. Permutational multivariate analysis of variance revealed changes in β-diversity according to dietary patterns. In multivariable-adjusted models, the adherence to the vege pattern was positively correlated with α-diversity. This is the first study to reveal a correlation between intestinal microbiota and dietary habits rich in vegetables and fruits in a rural area of Japan.

## 1. Introduction

The human gastrointestinal tract contains approximately 100 trillion bacteria, which have over three million gene functions. Intestinal bacteria affect the physiological functions of the host by producing thousands of metabolites and cell wall components that complement the functions of the host [1,2,3]. For example, short-chain fatty acids (SCFA) and neurotransmitters, which are characteristically produced by intestinal bacteria, contribute to regulating immune/metabolic functions and neural activity, respectively [4,5,6,7]. In correlation to these findings, many studies showed that patients with inflammatory bowel disease (IBD), obese individuals, and depression patients had different intestinal microbiota compared to healthy individuals [8,9,10].

The regulation of the intestinal microbiota is attributable primarily to diet [11]. Many findings reported on the mechanisms by which the ingredients affect intestinal microbiota. Dietary fiber, abundant in vegetables and fruits, serves as an energy source for intestinal bacteria and changes their composition. The metabolism of dietary fiber by intestinal bacteria leads to the production of essential metabolites, such as SCFA, which has been the target of many studies [12,13,14]. In addition, nutritional components in vegetables and fruits that affect the health conditions of the host via the intestinal microbiota include indigestible carbohydrates, such as oligosaccharides, and phytochemicals, such as polyphenols [11].

However, some studies investigating the association between diet and disease via the intestinal microbiota showed inconsistent results between the cases evaluating the intake of individual nutritional components and those of multiple food items. For example, animal studies showed that the development and progression of prostate cancer (PCa), which is known to be affected by intestinal bacteria, were inhibited by vitamins and isoflavones. Still, the efficacy of these nutrients was not observed in humans [15]. On the other hand, a cohort study found that those consuming a high-sugar, high-fat, and high-additives diet (Western Pattern, with low vitamins and isoflavones) had an increased risk of PCa, while those consuming a high vegetables, fruits, soy, and fish diet (Prudent Pattern, with high vitamins and isoflavones) had a decreased risk of PCa [15]. The inconsistent results may be because human dietary habits are generally rich in diversity, and the effects of the diet as a whole mask the effects of individual nutrients. In addition, some reports also showed that dietary habits rich in vegetables and fruits, similar to Prudent Pattern, improved human health and prevented chronic diseases [16,17,18]. Given the above, evaluating the diet from a macroscopic perspective is practical for a comprehensive assessment of health conditions.

The studies about intestinal microbiota were the same case. From a microscopic perspective, many studies reported the correlations between certain bacteria and disease [9,19,20,21,22]. However, no specific bacterial species is commonly correlated with overall human diseases. For example, obese patients were reported to have a higher proportion of Firmicutes and a lower proportion of Bacteroidetes [23], but opposite results were obtained in IBD patients [24,25]. This is likely because the health conditions are maintained by keeping the delicate balance of each physiological function. Conversely, the balance is disrupted by possessing many bacterial species that regulate certain physiological processes [26]. To analyze the effect of the intestinal microbiota from a macroscopic perspective, two diversity indices of the intestinal microbiota have been frequently used in recent studies. One is α-diversity, which quantifies the richness of the types of bacteria and the evenness of their proportions within an individual. In this index, a higher score indicates higher diversity of microbiota. The other is β-diversity, which evaluates the extent of difference in the types and proportions of the intestinal microbiota between individuals (or between groups) as a distance matrix. In this index, greater distance indicates the greater difference between samples in the intestinal microbiota. In evaluations of the diversity of intestinal microbiota and various diseases, it was reported that many disease patients, for instance, lifestyle-related diseases, inflammatory diseases, mental disorders and infections, had a decreased α-diversity compared to healthy individuals [27,28,29,30,31,32,33,34,35,36], suggesting that the richness and evenness of intestinal microbiota have important roles on not only specific diseases but also general health conditions.

This is likely because a decrease in α-diversity of intestinal microbiota causes a decline in the complementary/alternative functions of intestinal bacteria, making hosts vulnerable to changes in the external environment and susceptible to diseases [1].

Taking the above reports together, it is necessary to analyze the association between diet and intestinal microbiota from a macroscopic perspective, such as dietary habits and the diversity of the intestinal microbiota, rather than nutrients and certain bacteria to comprehensively capture human health conditions. Additionally, dietary habits rich in vegetables and fruits and an increased α-diversity of intestinal microbiota commonly has a positive effect on various disease. However, it is not clear whether dietary habits rich in vegetables and fruits have associated with α-diversity in Japan.

Therefore, the present study analyzed the effects of dietary habits on the diversity of intestinal microbiota in the Japanese population. A previous study comparing the dietary habits in Japan and Poland using factor analysis reported a marked difference in the dietary habits between the two countries [37]. Japanese people had different dietary habits that mix a traditional diet rich in soy and seaweeds with a Westernized diet [38], suggesting that the classifications based on Western dietary habits, such as the “Western diet [39]” and the “Mediterranean diet [40]”, are not appropriate in Japan. Therefore, the present study uniquely defined dietary habits in two ways and analyzed the correlations between dietary habits and the diversity of intestinal microbiota. The first way was “Dietary Pattern”, which was defined by factor analysis with reference to the previous studies [41,42,43]. The other way was SERVING SIZE, which was based on the “Dietary Balance Guide” established by the Ministry of Health, Labor and Welfare and the Ministry of Agriculture, Forestry, and Fisheries of Japan.

## 2. Materials and Methods

### 2.1. Study Design and Individuals 

In this study, the data of the Iwaki Health Promotion Project (2017) [44] were used to obtain basic information, such as gender and age from questionnaires, blood antioxidant levels from blood tests, the composition of the intestinal microbiota from stool sample tests, and dietary information from the BDHQ for analysis. Participants were males and females aged 20 years and older living in a rural area in northern Japan (Hirosaki City in Aomori Prefecture).

**Figure 1:**
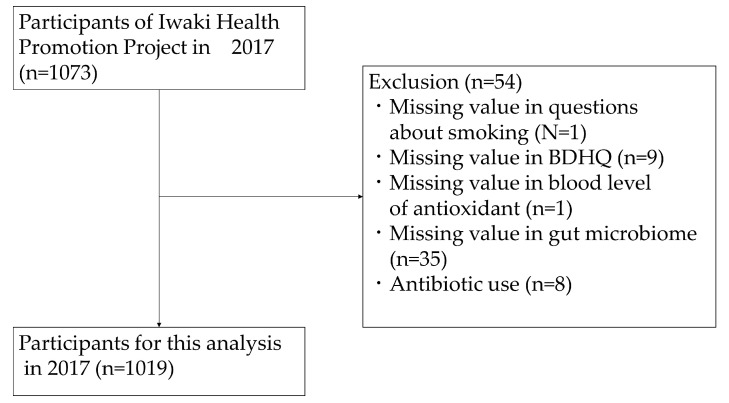
Individuals for the analysis.

A total of 1073 individuals underwent the project. After excluding those missing question items about the presence or absence of smoking history (one person), missing BDHQ data (nine people), missing data of blood antioxidant levels (one person), missing intestinal microbiota data (35 people), and responding “Yes” to a history of antibiotic use (eight people), 1019 individuals were selected for analysis (Figure 1).

This study was conducted with the approval of the ethics committees of the School of Medicine at Hirosaki University (2016-028) and KAGOME CO., LTD. (Tochigi, Japan) (2017-R06) following the principles of the Declaration of Helsinki. Informed consent was obtained from all participants before the study. This study has been registered in a public information database (UMIN-CTR, https://www.umin.ac.jp (accessed on 24 April 2023), UMIN ID: UMIN000040459).

### 2.2. Microbiota Analysis

Intestinal microbiota data were obtained according to the procedure described in a previous report [9]. The individuals’ stool samples were collected three to one day before the health checkup. DNA was extracted from the bead-treated fecal suspension using the automatic nucleic acid extraction system (Precision System Science, Chiba, Japan). The MagDEA DNA 200 (GC) reagent kit (Precision System Science) was used for automatic nucleic acid extraction. DNA extraction of all samples was completed within four months.

To amplify the V3-V4 region of the 16S rRNA gene, a universal primer set was used, according to a previous report [45]. Solution adjustment and condition setting for PCR amplification were carried out according to the same report [45]. PCR fragments purified using the PCR Cleanup Filter Plates (Merck Millipore, Burlington, MA, USA) were quantified by real-time quantitative PCR (qPCR) as previously reported [45]. To read the DNA sequences, purified PCR fragments were analyzed by 2 × 300 cycles of paired-end sequencing on the MiSeqTM system (Illumina, San Diego, CA, USA).

Paired-end reads were processed as follows: adapter sequences and low-quality bases (Q < 20) at the 3′ ends of the reads were trimmed with Cutadapt (version: 1.13) [46]. Reads containing ambiguous base N or shorter than 150 bases were excluded. Paired-end reads that satisfied the conditions were merged into one read, called a “merge read.” Merge reads shorter than 370 base pairs or longer than 470 base pairs were excluded with the fastq_mergepairs subcommand of VSEARCH (version: 2.4.3) [47]. Additionally, merge reads with one or more confirmed sequencing errors were excluded. After removing the chimeric reads detected with the uchime_denovo subcommand of VSEARCH, the remaining merge reads were clustered with a minimum sequence homology of 97% to obtain an operational taxonomic unit (OTU). Phylogenetic prediction for OTUs was performed by applying RDP Classifier (commit hash: 701e229dde7cbe53d4261301e23459d91615999d) [48] based on its representative reads. The ones whose reliability of prediction result was 0.8 or lower were treated as unclassified. The relative abundance of each bacterial genus in the intestinal microbiota was calculated by dividing the number of read counts for each bacterial genus by the total number of read counts.

In addition, we defined the proportion of individuals with more than one read count of a bacteria genus as Share Rating (for instance, if four of ten individuals have more than one read count of a bacteria genus, the Share Rating of the bacteria genera is 40%), and intestinal bacteria genera with less than 1% Share Rating were excluded for analysis.

### 2.3. Dietary Assessment and Dietary Pattern Definition

The BDHQ was used to estimate the intake of approximately 30 types of nutrients and approximately 50 food/beverage items over the past month [49]. The individuals’ answered the BDHQ at home 2–3 weeks before stool sample collection, and the responses were checked and collected on the day of the health checkup by researchers. The responses were analyzed by the DHQ Support Center of Gender Medical Research CO., LTD., and the intake of each food item was calculated [49]. From the result, the intake of each food group was calculated according to the classification of 15 food groups defined by the BDHQ developers (http://www.nutrepi.m.u-tokyo.ac.jp/english/dhq/dhq.html (accessed on 24 April 2023), Table 1). Next, the data of food group intake was adjusted for total energy intake using a correction method called the residual method.

As indices for evaluating overall dietary habits, two methods were adopted: (1) dietary pattern and (2) serving size.

(1) Dietary Pattern: To derive dietary patterns, previous studies were referred [41,42,43]. Briefly, the Kaiser-Meyer-Olkin (KMO) test was performed to determine whether each observed factor (each food group in this study) has at least one latent factor present and whether the dataset has at least one latent factor. As a result, two food groups, SWT (Sweets) and BEV (Beverage), were excluded based on measures of sampling adequacy (MSA) for each item > 0.2, and the dataset excluding SWT and BEV was confirmed to have at least one latent factor based on overall MSA > 0.7. The number of latent factors was determined based on the parallel analysis by scree plot, which is an analytical method that compares the eigenvalues of the latent factors in the used dataset with that in the random dataset. As a result, six latent factors were suggested. Using factor analysis, the factor loadings, which is the proportion of each food group contributing to each latent factor, were calculated by the minimum residue method under varimax rotation conditions. The factor scores for each participant were calculated using all food groups, regardless of loading. The dietary patterns were named based on food groups with high factor loadings for each latent factor. Adherence to the dietary patterns was divided into quartiles, with Quartile 1 (Low factor scores) representing the lowest adherence and Quartile 4 (High factor scores) representing the highest adherence to the pattern.

(2) Serving Size: Serving size was defined in the “Dietary Balance Guide”, which represents a healthy diet to prevent lifestyle-related diseases, stipulated by the Ministry of Health, Labor and Welfare and the Ministry of Agriculture, Forestry, and Fisheries of Japan (https://www.mhlw.go.jp/bunya/kenkou/pdf/eiyou-syokuji4.pdf (accessed on 24 April 2023)). In the Dietary Balance Guide, the dishes in a meal were divided into five categories: staple food (grain-based dishes), side dish (vegetables, mushroom, potato, and seaweeds-based dishes), main dish (meat, fish, egg, and soy-based dishes), dairy products, and fruits. Each category has a serving size that indicates the ideal amount that should be consumed daily. The score of serving size was calculated based on the “Calculation method of Serving Size of the Dietary Balance Guide in BDHQ”, described in DHQ Support Center, as well as “nutrition survey report using BDHQ”, described in Odawara City in Kanagawa Prefecture. Briefly, individuals received a full score for serving size in each category if their intakes of each category met the defined ideal amount. If not, the score was deducted according to the amount of excess or deficient. The final serving size score was calculated as the total score of each category. Adherence to serving size was divided into quartiles, with Quartile 1 (low scores) representing the lowest adherence and Quartile 4 (high factor scores) representing the highest adherence to serving size.

### 2.4. Other Measurements

Data on age, gender, smoking history (yes/no/not currently), drinking history (yes/no/not currently), and history of antibiotic use (yes/no) were collected using the questionnaire. In analyzing, the answer “not currently” was included in that of “no”. Clinical characteristics, such as height, weight, and body mass index (BMI), were all collected by the Department of Social Medicine in the Hirosaki COI Research Promotion Organization.

### 2.5. Statistical Analysis

In analyzing β-diversity, two indices (Bray–Curtis distance and Jaccard distance) were adopted. Permutational multivariate analysis of variance (PERMANOVA) was performed by setting the relative abundance of intestinal bacteria at the genus level as the outcome and the quartile of each dietary pattern score as the exposure. Age, gender, smoking and drinking habits, and BMI were used as covariates.

In analyzing α-diversity, four indices (Shannon, Pielou, Simpson, and Invsimpson) were adopted. The Shannon index accounts for both the abundance and evenness of the bacteria genera present. The Pielou index accounts for only the evenness of the bacteria genera present. The Simpson and Invsimpson indices are similar to the Shannon and Pielou indices, respectively. The difference is that dominant species are given more weight in the calculation of the Simpson and Invsimpson index. Multiple regression analysis was performed by setting the score of each α-diversity index as the outcome and the quartile of each dietary pattern score as the exposure. Age, gender, smoking and drinking habits, and BMI were used as covariates.

In analyzing individual genera, multiple regression analysis was performed by setting the relative abundance of intestinal bacteria genera as the outcome and the quartile of each dietary pattern score as the exposure. Age, gender, smoking and drinking habits, and BMI were used as covariates.

As for each variable, the quartile of each dietary pattern score was treated as continuous variables, and the significant difference test was calculated as *p* for trend. BMI, scores of α-diversity indices, and relative abundance of intestinal microbiota were Z-scored and treated as continuous variables. Age (by age group), gender, and smoking and drinking habits were treated as categorical variables. R version 4.0.3 and the packages of GPArotation and vegan were used for analysis.

## 3. Results

### 3.1. Summary Statistics of Data

A total of 1019 individuals, with a mean age ± SD of 55 ± 15 years, were included in the analysis. Females accounted for 58.4% of the individuals, and the smoking and drinking rates were 14.8% and 47.2%, respectively. The mean BMI ± SD was 23.1 ± 3.6 kg/m^2^ (Table 2).

### 3.2. Definition of Dietary Patterns

**Figure 2:**
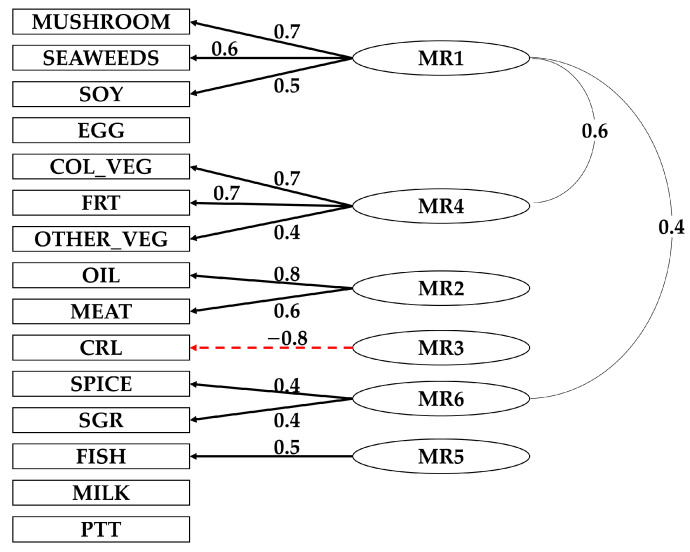
Estimation of dietary patterns by factor analysis. Six factors (dietary patterns) were estimated by factor analysis using 15 food groups of BDHQ data. Each factor is named MR after it was estimated by Minimum Residual Method. Black solid lines indicate a positive correlation. Red dashed lines indicate a positive correlation. Numerical values represent correlation coefficients. (COL_VEG: Colored Vegetables, FRT: Fruits, OTHER_VEG: Other Vegetables, CRL: Cereals, SGR: Sugar, PTT: Potato).

**Figure 3:**
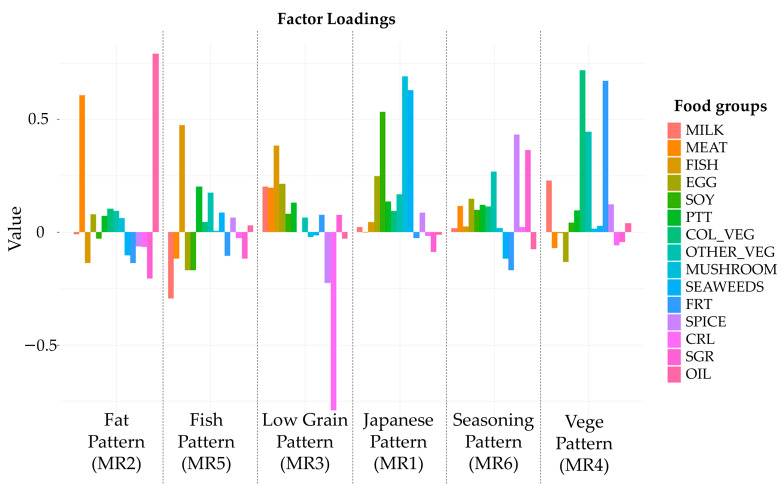
Contribution of food groups to each dietary pattern. The contribution of 15 food groups to each dietary pattern (MR1~MR6) was shown as factor loading. (COL_VEG: Colored Vegetables, FRT: Fruit, OTHER_VEG: Other Vegetables, CRL: Cereals, SGR: Sugar, PTT: Potato).

Six factors were determined by factor analysis, and the correlations between the factors (dietary patterns) and their constituent variables (food groups) are shown in Figure 2. The MRs indicated factors estimated by factor analysis (they were named MRs as they were estimated by the Minimum Residual Method), and the examination of inter-factor correlations identified positive correlations between MR1 and MR4 and between MR1 and MR6. Although factors often had positive correlations with their constituent variables, MR3 was found to have a negative correlation with its variables, cereals. In addition, Figure 3 shows the results of factor loadings. Each factor was found to have large-load variables with a factor loading of >|0.5|. Focusing on their large-load variables, the six factors were named Japanese Pattern (MR1; mushroom, seaweeds, and soy), Vege Pattern (MR4; colored vegetables, other vegetables, and fruits), Fat Pattern (MR2; meat and oil), Low Grain Pattern (MR3; cereals), Seasoning Pattern (MR6; seasoning spices and sugar), and Fish Pattern (MR5; fish) and used for subsequent analyses. However, because MR6 had no variables with a factor loading of >|0.5|, we focused on two variables with high factor loadings.

### 3.3. Summary Statistics of Dietary Pattern Quartiles

Table 2 showed the basic statistics for age, BMI, sex, smoking, and drinking of participants divided into quartiles according to the scores of six dietary patterns or serving sizes. There were statistically significant differences in various elements between quartiles of the Vege Pattern. The population with the higher factor scores of Vege Pattern had significantly higher age and proportions of females, non-smokers, and non-drinkers than the population with the lower, and there was no association with BMI. The trend observed for Vege Pattern was also observed for serving size. In addition, among other dietary patterns, the Japanese Pattern notably showed a similar trend as Vege Pattern. Additionally, among all dietary patterns, only the Fat Pattern showed significantly lower age with a higher factor score of the dietary pattern.

### 3.4. Dietary Pattern and β-Diversity

Table 3 showed the association between the six dietary patterns or serving sizes divided into quartiles and β-diversity (Bray–Curtis distance and Jaccard distance evaluated by PERMANOVA). Both Bray–Curtis distance and Jaccard distance showed significant differences in all dietary patterns and serving sizes, except Seasoning Pattern and Fish Pattern.

### 3.5. Dietary Pattern and α-Diversity

**Figure 4:**
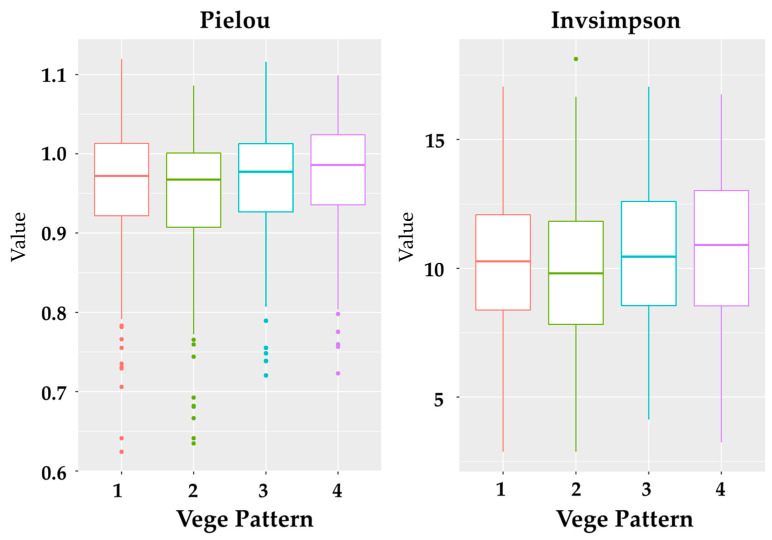
The boxplot of the Pielou (**left**) and the Invsimpson (**right**) diversities in each quartile of the Vege Pattern. Plots show outliers greater than the mean ± 1.5 × range from Quartile 1 to Quartile 4.

Table 4 showed the association between the six dietary patterns or serving sizes divided into quartiles and α-diversity. Only the Vege Pattern showed a statistically significant association with the indices of α-diversity (Pielou and Invsimpson). Figure 4 showed that these α-diversity indices had a positive correlation with the score of the Vege Pattern.

### 3.6. Food Group and α-Diversity

To investigate the food groups and food items contributing to the increase in α-diversity in the Vege Pattern, we examined the association between each food group/item and α-diversity. Table 5 showed the correlations between the food groups and α-diversity. All food groups (COL_VEG, OTHER_VEG, and FRT) showed no correlation with α-diversity.

### 3.7. Food Items and α-Diversity

Next, the correlations between food items and α-diversity were shown in Table 6. Green and yellow vegetables (GrYwVege) showed a positive correlation with an index of α-diversity (Pielou), and citrus and persimmons and strawberry (PerStr) positively correlated with all four indices of α-diversity.

### 3.8. Association between Individual Bacterial Genera and Dietary Patterns

We investigated the bacterial genera with fluctuations in the following four elements that showed a correlation with α-diversity: dietary pattern “Vege Pattern”, as well as food items “GrYwVege”, “Citrus”, and “PerStr”. The bacterial genera in which the *p* for trend of each element was 0.05 or less are shown in Supplemental Table S1. The numbers of bacterial genera detected to be significantly associated with the Vege Pattern, GrYwVege, Citrus, and PerStr were 12, 11, 15, and 14, respectively, and overall 40 bacterial genera were detected. Among those, nine bacterial genera were detected by multiple elements, and they all showed correlations in the same direction (positive and negative, Table 7).

### 3.9. Limitations

Although the intake of each food item in BDHQ was adjusted by the residual method, these intakes might be overestimated or underestimated due to the nature of data acquisition of BDHQ, which may have affected the results [50]. In addition, age, gender, smoking and drinking habits, and BMI were considered covariates, but the involvement of other confounding factors, such as income and exercise, might not have been eliminated. Due to the nature of the sample collection, the results of this study applied only to males and females aged 20 years and older living in a rural area in northern Japan (Hirosaki City in Aomori Prefecture). Thus, it is unclear whether the defined dietary patterns can be applied in other areas.

## 4. Discussion

Various definitions of dietary patterns have been used to evaluate the effects of diet on intestinal microbiota in Western countries. A factor analysis of the Food Frequency Questionnaire (FFQ) of 517 elderly males in the United States defined two patterns: the Western pattern, which is rich in meat, cereals, potato, egg, and snacks, and the Prudent pattern, which is rich in fruits, vegetables, soy, fish, and chicken meat [41]. In the present study, factor analysis was performed using Japanese data with reference to this previous report [41], and six factors were defined from BDHQ data (Figure 2). As a result, the previous study showed no correlation between dietary patterns and intestinal microbiota, while the present study clarified that some dietary patterns were correlated with the composition of the intestinal microbiota (Table 3 and Table 4).

In this study, we analyzed the association between dietary habits and intestinal microbiota from the viewpoint of diversity. Our results showed that the β-diversity of the intestinal microbiota was significantly correlated with the four dietary patterns, Japanese Pattern, Vege Pattern, Fat Pattern, and Low Grain Pattern (Table 3). Furthermore, the score of the Vege Pattern had a positive correlation with the α-diversity of the intestinal microbiota (Table 4). The fluctuation in α-diversity was not observed in the other five dietary patterns. These results revealed that although intestinal microbiota was likely to change according to dietary patterns, α-diversity was increased only by the dietary pattern with a high intake of vegetables and fruits. Similar results were reported by a study conducted in Greece, where a dietary habit rich in vegetables and fruits increased α-diversity while other dietary habits did not [51]. Whereas the score of serving size was significantly correlated with β-diversity but not α-diversity of the intestinal microbiota. This suggested that the dietary pattern could be a better indicator to capture the fluctuation of intestinal microbiota than serving size. This difference might be due to the calculation method of serving size, which treated all categories of dishes equally and added them together as the total score.

The score of Vege Pattern had a positive correlation with Pielou and Invsimpson diversities, which are α-diversity indices emphasizing the “evenness” of the types of intestinal bacteria, not Shannon and Simpson diversities, which are α-diversity indices considering both “abundance” and “evenness” of the types of intestinal bacteria (Table 4). These results suggest that the dietary pattern rich in vegetables and fruits reduces the exclusive bacterial species, rather than increasing the intestinal bacteria species. However, there was no correlation between the intake of vegetables or fruits alone and α-diversity (Table 5), suggesting that a mixed intake of both vegetables and fruits was necessary to increase α-diversity. It was shown that the intake of green and yellow vegetables, citrus, and persimmon/strawberry might be involved in the fluctuations of α-diversity (Table 6).

The Mediterranean diet, which is a diet rich in fruits, vegetables, olive oil, nuts, beans, and whole grains containing high polyphenols, monounsaturated fatty acids, polyunsaturated fatty acids, dietary fiber, and vegetable proteins, has also been reported as a dietary habit increasing α-diversity [52,53]. In addition, it was reported that the Mediterranean diet contributed to a decrease in the relative abundance of *Escherichia coli*, an increase in SCFA-producing bacteria, and an accompanying increase in fecal SCFA content [52]. In the present study, the high adherence to Vege Pattern also showed a decrease in *Escherichia Shigella*, which is closely related to *Escherichia coli*, and an increase in *Faecalibacterium*, which is major SCFA-producing bacteria (Table 7). Conversely, dietary habits that decrease α-diversity include the ketogenic diet (low sugar and high fat) and the Western diet (high sugar and high fat) [51]. Still, our analysis did not detect any dietary patterns with a significant decrease in α-diversity of the intestinal microbiota (Table 4). This may be because the Fat Pattern and the Low Grain Pattern variance in the Japanese population are smaller than in the Western population, and those with a high or low intake of carbohydrates and fat may not be classified appropriately compared to Western countries.

As mentioned in the introduction section, an increased α-diversity has a positive association with various health conditions. Thus, dietary patterns rich in vegetables and fruits may have positive associations with health conditions via an increased α-diversity. Additional analyses are necessary to clarify these associations in Japanese data. Furthermore, vegetables and fruits have a lot of nutrients for improving health conditions, for instance, carotenoids and dietary fibers. Thus, it is necessary to analyze if the effect of these foods on health conditions act directly by original nutrients or indirectly via an increased α-diversity.

The increased α-diversity in the Vege Pattern is likely because each bacterial genus was affected. For example, nine bacterial genera commonly exhibited a change in four elements that increased α-diversity (Vege Pattern, GrYwVege, Citrus, and PerStr, Table 6). Among those, *Barnesiella*, *Actinomyces*, *Faecalibacterium*, *Escherichia-Shigella*, and *Solobacterium* had a high share rating (30% or more), and they likely have contributed to the changes in intestinal microbiota due to the diet rich in vegetables and fruits. In addition, some of these bacterial genera were previously reported to be associated with consuming vegetables and fruits. In a study investigating the association between daily intake of vegetable/fruit juice and the fluctuations in the intestinal microbiota, a significant increase in α-diversity of the intestinal microbiota and an increase in the relative abundance of *Faecalibacterium* were observed after three weeks of juice intake [54]. The relative abundance of SCFA-producing bacteria, such as *Faecalibacterium*, was reported to increase in type 2 diabetic patients that were intervened with a therapeutic diet rich in dietary fiber, which is abundant in vegetables [55]. Additionally, *Faecalibacterium* was reported to be associated with disease, and a decrease in *Faecalibacterium* was reported in patients with Crohn’s disease [56]. Concerning its mechanism, *Faecalibacterium* was reported to induce anti-inflammatory effects [56]. As for *Escherichia-Shigella*, which was negatively associated with the Vege Pattern, a vegetable diet was negatively correlated with *Escherichia-Shigella* in a randomized controlled trial using dogs [57].

In this study, we found that the score of Vege Pattern was correlated with α-diversity indices with an emphasis on “evenness” (Pielou and Invsimpson diversities), not α-diversity indices with an emphasis on “abundance” and “evenness” (Shannon and Simpson diversities, Table 4). Although few studies have focused on the difference between “abundance” and “evenness” and analyzed their association with health conditions, α-diversity has been reported to positively correlate with health conditions regardless of whether the emphasis of α-diversity lies in the viewpoint of “abundance” or “evenness” [58,59,60]. Additionally, many have reported a positive correlation between the increase in SCFA-producing bacteria and improved health conditions. Taking these reports and the results of the present study together, the increases in α-diversity and SCFA-producing bacteria due to a diet rich in vegetables and fruits are thought to be involved in the maintenance and improvement of health conditions.

## 5. Conclusions

This study serves to elucidate the association between diet and intestinal microbiota from a macroscopic perspective, such as dietary patterns and diversities of intestinal microbiota. Using factor analysis, we defined six kinds of dietary patterns from the dietary questionnaire of 1019 individuals in the Iwaki Health Promotion Project. This study is the first report in Japan that showed that increased α-diversity of microbiota was found only in the dietary pattern with a high intake of vegetables and fruits. This fluctuation of intestinal microbiota is possibly involved in maintaining and improving health conditions. In the future, it is essential to clarify the association between dietary patterns and health conditions via intestinal microbiota.

## Figures and Tables

**Table 1:** Fifteen food groups consisting of food items.

Food Group	Food Item
MUSHROOM	Mushroom
SEAWEEDS	Seaweeds
SOY	Tofu, Natto
EGG	Egg
COL_VEG (Colored Vegetables)	Green & Yellow Pickles, Green & Yellow Vegetables, Carrot & Pumpkin, Tomato
FRT (Fruits)	Citrus, Persimmon & Strawberry, Other Fruits, Fruit Juice
OTHER_VEG (Other Vegetables)	Other Pickles, Raw Lettuce & Cabbage, Cabbage, Radish, Root Vegetables
OIL	Cooking Oil
MEAT	Chicken, Pork & Beef, Ham, Lever
CRL (Cereals)	Bread, Soba, Udon, Ramen, Pasta, Rice
SPICE	Mayonnaise, Miso Soup, Noodle Soup, Soy Sauce, Cooking Salt
SGR (Sugar)	Sugar, Cooking Sugar
FISH	SOSS (Squid, Octopus, Shrimp, Shellfish), Bone Fish, Tuna, Dried Fish, Greasy Fish, Less Fat Fish
MILK	Low Fat Milk, Milk, Ice-cream
PTT (Potato)	Potato
SWT (Sweets)	Pastry, Japanese Sweets, Rice Cracker
BEV (Beverage)	Green Tea, Black Tea, Coffee, Coke, Sake, Beer, Shochu, Whiskey, Wine

**Table 2:** Overall participant attributes according to dietary pattern quartile.

Charasteristics		n	Age	BMI	Sex	Smoking	Drinking
					Female (%)	Yes (%)	Yes (%)
Overall		1019	54.7 ± 15.1	23.1 ± 3.6	58.4	14.8	47.2
Japanese pattern	1 (Lowest)	255	51.9 ± 14.4	23.7 ± 3.8	34.5	23.9	56.5
	2	254	53.7 ± 15.0	23.0 ± 3.6	60.6	14.6	48.4
	3	255	54.5 ± 15.3	22.7 ± 3.7	69.4	11.8	43.5
	4 (Highest)	255	58.9 ± 15.0	22.9 ± 3.3	69.0	9.4	40.4
	*p* value		**<0.0001**	**0.0150 **	**<0.0001**	**<0.0001**	**0.0018 **
Vege pattern	1 (Lowest)	254	51.7 ± 14.3	23.5 ± 3.7	34.6	22.8	63.4
	2	255	51.6 ± 15.8	22.9 ± 3.8	62.0	14.5	45.5
	3	255	55.1 ± 15.1	22.9 ± 3.6	69.4	12.2	39.6
	4 (Highest)	255	60.6 ± 13.6	22.9 ± 3.4	67.5	10.2	40.4
	*p* value		**<0.0001**	0.1450	**<0.0001**	**0.0003 **	**<0.0001**
Fat pattern	1 (Lowest)	255	60.0 ± 14.2	23.4 ± 3.5	45.9	13.7	52.9
	2	255	56.8 ± 14.0	22.8 ± 3.3	65.5	15.3	37.3
	3	255	52.7 ± 15.4	23.0 ± 3.5	61.6	15.3	51.4
	4 (Highest)	254	49.5 ± 14.8	23.1 ± 4.1	60.6	15.4	47.2
	*p* value		**<0.0001**	0.3630	**<0.0001**	0.9442	**0.0016 **
Low grain pattern	1 (Lowest)	255	53.5 ± 14.7	23.7 ± 3.9	35.3	22.0	51.0
	2	255	54.9 ± 15.4	23.0 ± 3.4	62.0	11.0	42.0
	3	254	55.0 ± 14.6	22.7 ± 3.5	71.3	10.6	42.9
	4 (Highest)	255	55.6 ± 15.8	22.9 ± 3.5	65.1	16.1	52.9
	*p* value		0.4740	**0.0229 **	**<0.0001**	**0.0007 **	**0.0232 **
Seasoning pattern	1 (Lowest)	254	54.6 ± 14.4	23.3 ± 3.9	46.1	15.7	52.8
	2	255	55.3 ± 14.3	23.1 ± 3.4	62.7	14.9	44.3
	3	255	53.5 ± 15.5	22.6 ± 3.5	64.7	14.5	47.1
	4 (Highest)	255	55.7 ± 15.3	23.3 ± 3.7	60.0	14.5	44.7
	*p* value		0.3910	0.1110	**<0.0001**	0.9768	0.2004
Fish pattern	1 (Lowest)	255	51.9 ± 14.3	23.1 ± 3.9	45.1	17.6	52.9
	2	255	52.8 ± 15.0	23.0 ± 3.5	60.8	18.0	48.6
	3	255	54.9 ± 14.6	22.9 ± 3.7	62.4	13.3	41.6
	4 (Highest)	254	59.4 ± 15.7	23.3 ± 3.4	65.4	10.6	45.7
	*p* value		**<0.0001**	0.7400	**<0.0001**	0.0541	0.0699
SERVING SIZE	1 (Lowest)	255	50.9 ± 14.6	22.7 ± 3.3	46.3	23.1	65.9
	2	254	53.0 ± 15.5	23.4 ± 3.7	51.6	13.0	55.1
	3	255	56.3 ± 15.2	23.3 ± 3.7	62.0	15.3	40.4
	4 (Highest)	255	58.8 ± 14.1	23.0 ± 3.7	73.7	8.2	27.5
	*p* value		**<0.0001**	0.0994	**<0.0001**	**<0.0001**	**<0.0001**

Each numerical variable (Age, BMI) is expressed as mean ± SD, and each categorical variable (Sex, Smoking, Drinking) is expressed as a percentage. Orange letters indicate a positive correlation. Blue letters indicate a negative correlation. *p*-value was used for the comparison among four quartiles for each dietary pattern (Quatile 1 to 4). *p*-value was calculated by ANOVA (numerical variables) and χ2 test (categorical variables). Multiple testing correction was performed by the Bonferroni method.

**Table 3:** Association between dietary patterns and β-diversity.

Beta Diversity		Japanese Pattern	Vege Pattern	Fat Pattern	Low Grain Pattern	Seasoning Pattern	Fish Pattern	SERVING SIZE
*p* for trend	Bray	**0.001**	**0.027**	**0.001**	**0.028**	0.086	0.106	**0.001**
	Jaccard	**0.001**	**0.030**	**0.001**	**0.047**	0.122	0.114	**0.001**

*p* for trend was calculated by PERMANOVA. Covariates: Age + Gender + Smoking + Drinking + BMI. The bold: *p* for trend < 0.05.

**Table 4:** Association between dietary patterns and α-diversity.

Alpha Diversity		Shannon	Pielou	Simpson	Invsimpson
Japanese Pattern	β coefficient	−0.070	−0.056	−0.017	−0.055
	95% CI	−0.153	−0.139	−0.101	−0.139
		0.013	0.028	0.067	0.029
	*p* for trend	0.100	0.190	0.693	0.197
Vege Pattern	β coefficient	0.046	0.103	0.056	0.084
	95% CI	−0.029	0.028	−0.020	0.009
		0.121	0.179	0.131	0.160
	*p* for trend	0.231	**0.007**	0.150	**0.029**
Fat Pattern	β coefficient	0.023	0.008	0.037	0.016
	95% CI	−0.040	−0.055	−0.026	−0.047
		0.086	0.071	0.101	0.079
	*p* for trend	0.474	0.808	0.247	0.623
Low Grain Pattern	β coefficient	−0.027	−0.030	−0.023	−0.030
	95% CI	−0.090	−0.093	−0.086	−0.094
		0.036	0.034	0.041	0.034
	*p* for trend	0.396	0.361	0.485	0.353
Seasoning Pattern	β coefficient	0.040	0.019	−0.002	0.036
	95% CI	−0.026	−0.047	−0.068	−0.030
		0.105	0.085	0.065	0.102
	*p* for trend	0.238	0.570	0.965	0.289
Fish Pattern	β coefficient	0.053	0.022	0.015	0.025
	95% CI	−0.006	−0.038	−0.046	−0.035
		0.113	0.082	0.075	0.085
	*p* for trend	0.079	0.468	0.634	0.410
SERVING SIZE	β coefficient	−0.027	0.034	0.029	0.026
	95% CI	−0.086	−0.025	−0.030	−0.033
		0.031	0.092	0.088	0.085
	*p* for trend	0.356	0.260	0.335	0.385

*p* for trend was calculated by multiple regression analysis. Covariates: Age + Gender + Smoking + Drinking + BMI. The bold: *p* for trend < 0.05.

**Table 5:** Association between food groups and α-diversity.

Alpha Diversity		Shannon	Pielou	Simpson	Invsimpson
COL_VEG	β coefficient	0.017	0.052	0.016	0.046
	95% CI	−0.054	−0.020	−0.056	−0.026
		0.089	0.124	0.088	0.118
	*p* for trend	0.640	0.159	0.662	0.210
OTHER_ VEG	β coefficient	0.040	0.009	0.013	0.019
	95% CI	−0.030	−0.062	−0.058	−0.052
		0.110	0.080	0.083	0.089
	*p* for trend	0.265	0.804	0.728	0.608
FRT	β coefficient	0.001	0.035	0.036	0.021
	95% CI	−0.063	−0.030	−0.029	−0.044
		0.064	0.099	0.100	0.085
	*p* for trend	0.986	0.289	0.278	0.528

*p* for trend was calculated by multiple regression analysis. Covariates: Age + Gender + Smoking + Drinking + BMI. (COL_VEG: Colored Vegetables, OTHER_VEG: Other Vegetables, FRT: Fruit).

**Table 6:** Association between food items and α-diversity.

Alpha Diversity		Shannon	Pielou	Simpson	Invsimpson
GrYwPick	β coefficient	0.000	−0.004	−0.024	−0.025
	95% CI	−0.056	−0.061	−0.081	−0.082
		0.057	0.053	0.033	0.032
	*p* for trend	0.986	0.890	0.411	0.387
GrYwVege	β coefficient	0.053	0.057	0.039	0.053
	95% CI	−0.002	0.002	−0.017	−0.003
		0.108	0.113	0.095	0.109
	*p* for trend	0.059	**0.043**	0.168	0.061
CarPump	β coefficient	−0.035	−0.015	−0.019	−0.025
	95% CI	−0.092	−0.072	−0.077	−0.083
		0.021	0.042	0.038	0.032
	*p* for trend	0.221	0.604	0.507	0.390
Tomato	β coefficient	0.016	0.039	0.047	0.029
	95% CI	−0.040	−0.017	−0.008	−0.027
		0.071	0.095	0.103	0.085
	*p* for trend	0.577	0.169	0.096	0.312
OtherPick	β coefficient	0.055	0.019	0.030	0.024
	95% CI	−0.002	−0.039	−0.027	−0.034
		0.112	0.077	0.088	0.082
	*p* for trend	0.060	0.519	0.303	0.421
RawLtCb	β coefficient	0.029	0.020	0.031	0.041
	95% CI	−0.026	−0.035	−0.024	−0.014
		0.083	0.075	0.086	0.097
	*p* for trend	0.305	0.476	0.273	0.142
Cabbage	β coefficient	0.001	0.002	−0.004	−0.003
	95% CI	−0.054	−0.054	−0.060	−0.059
		0.057	0.058	0.052	0.053
	*p* for trend	0.958	0.942	0.895	0.922
Radish	β coefficient	0.010	0.005	0.011	0.003
	95% CI	−0.046	−0.051	−0.046	−0.054
		0.066	0.062	0.067	0.059
	*p* for trend	0.734	0.856	0.714	0.928
RootVege	β coefficient	0.018	0.018	0.017	0.014
	95% CI	−0.037	−0.038	−0.039	−0.042
		0.074	0.075	0.074	0.071
	*p* for trend	0.519	0.522	0.545	0.621
Citrus	β coefficient	0.071	0.075	0.078	0.067
	95% CI	0.015	0.019	0.021	0.010
		0.127	0.132	0.134	0.124
	*p* for trend	**0.013**	**0.009**	**0.007**	**0.020**
PerStr	β coefficient	0.083	0.085	0.094	0.085
	95% CI	0.028	0.029	0.038	0.028
		0.139	0.141	0.150	0.141
	*p* for trend	**0.003**	**0.003**	**0.001**	**0.003**
OtherFruits	β coefficient	−0.038	0.004	−0.005	−0.022
	95% CI	−0.098	−0.055	−0.064	−0.082
		0.021	0.064	0.055	0.038
	*p* for trend	0.202	0.883	0.880	0.470
Juice	β coefficient	0.025	0.035	0.027	0.038
	95% CI	−0.030	−0.020	−0.029	−0.017
		0.080	0.091	0.082	0.094
	*p* for trend	0.379	0.210	0.345	0.177

*p* for trend was calculated by multiple regression analysis. Covariates: Age + Gender + Smoking + Drinking + BMI. (GrYwPick: Green and Yellow Pickles, GrYwVege: Green and Yellow Vegetables, CarPump: Carrot and Pumpkin, OtherPick: Other Pickles, RawLtCb: Raw Lettuce and Cabbage, RootVege: Root Vegetables, PerStr: Persimmon and Strawberry). The bold: *p* for trend < 0.05.

**Table 7:** Bacterial genera that fluctuated with elements correlated with α-diversity (Vege Pattern, GrYwVege, Citrus, and PerStr).

Genus	Vege Pattern	GrYw Vege	Citrus	PerStr	Share Rating (%)	Median Relative Abundance (%)
*Neisseria*	〇	〇	〇		3.2	0.0046
*Barnesiella*	〇	〇		〇	40.1	0.15
*Actinomyces*	●		●	●	97.0	0.052
*Faecalibacterium*	〇		〇		97.4	7.3
*Escherichia.Shigella*	●		●		77.9	0.037
*Solobacterium*	●			●	33.8	0.0071
*Acinetobacter*		●		●	1.7	0.0030
*Succinivibrio*			●	●	2.5	0.12
*Gardnerella*			●	●	1.4	0.011

*p* for trend was calculated by multiple regression analysis. Covariates: Age + Gender + Smoking + Drinking + BMI. Only nine bacterial genera detected in multiple elements are shown. Open circles indicate a positive correlation and filled circles indicate a negative correlation. Share Rating indicates the proportion of individuals with more than one read count of a bacteria genus. (GrYwVege: Green and Yellow Vegetables, PerStr: Persimmon and Strawberry).

## Data Availability

The data are not publicly available due to ethical concerns. Data are available from the Hirosaki University COI Program Institutional Data Access/Ethics Committee for researchers who meet the criteria for access to the data.

## Supplementary Materials

The following supporting information can be downloaded at: https://www.mdpi.com/article/10.3390/nu15092104/s1, Table S1: Bacterial genera that showed correlations with the intake of the Vege Pattern, GrYwVege, Citrus, and PerStr.
