# Supplementary material for: Definition of a Dietary Pattern Expressing the Intake of Vegetables and Fruits and Its Association with Intestinal Microbiota
Source: Nutrients. 2023 Apr 27;15(9):2104. doi: 10.3390/nu15092104 (PMC10181458; doi:10.3390/nu15092104)
Supplement: Supplementary file 1 [file nutrients-15-02104-s001.zip › nutrients-2295023-SI.pdf]

Supplemental Table S1. Bacterial genera that showed correlations with the intake of the Vege Pattern, GrYwVege, Citrus, and PerStr.

Vege Pattern	*P* for trend	beta	Share Rating (%)	Median relative abundance (%)
Mitsuokella	0.0006	0.000913	10.6	0.40
Barnesiella	0.0071	0.000277	40.1	0.15
Actinomyces	0.0137	-0.000094	97.0	0.052
Calditerricola	0.0188	0.000003	2.1	0.0052
Faecalibacterium	0.0206	0.004927	97.4	7.3
Atopobium	0.0248	-0.000046	40.1	0.022
Neisseria	0.0263	0.000001	3.2	0.0046
Escherichia.Shigella	0.0296	-0.001369	77.9	0.037
Lactonifactor	0.0317	-0.000010	31.6	0.010
Varibaculum	0.0386	-0.000018	3.4	0.0083
Solobacterium	0.0388	-0.000007	33.8	0.0071
Rothia	0.0485	-0.000026	59.5	0.014

GrYw Vege	*P* for trend	beta	Share Rating (%)	Median relative abundance (%)
Alloprevotella	0.0054	-0.0011301	14.2	0.97
Scardovia	0.0081	-0.0000022	5.9	0.0074
Acinetobacter	0.0145	-0.0000005	1.7	0.0030
Coprococcus	0.0191	0.0005298	74.6	0.56
Bacillus	0.0272	0.0001075	55.2	0.043
Barnesiella	0.0283	0.0001663	40.1	0.15
Asaccharobacter	0.0331	0.0000227	7.2	0.012
Proteus	0.0415	-0.0000040	4.1	0.0032
Kocuria	0.0440	-0.0000008	4.1	0.0061
Lactococcus	0.0456	0.0000583	40.9	0.0077
Neisseria	0.0467	0.0000009	3.2	0.0046

Citrus	*P* for trend	beta	Share Rating (%)	Median relative abundance (%)
Actinomyces	0.0080	-0.000076	97.0	0.052

Abiotrophia	0.0081	-0.000003	11.4	0.0054
Lachnospiracea_incertae_sedis	0.0097	0.000940	99.9	1.6
Allisonella	0.0118	-0.000032	41.0	0.031
Succinivibrio	0.0141	-0.000166	2.5	0.12
Neisseria	0.0199	0.000001	3.2	0.0046
Gardnerella	0.0214	-0.000002	1.4	0.011
Faecalibacterium	0.0221	0.003666	97.4	7.3
Tumebacillus	0.0229	0.000000	1.3	0.0030
Escherichia.Shigella	0.0279	-0.001039	77.9	0.037
Streptococcus	0.0310	-0.002261	100.0	0.80
Stomatobaculum	0.0339	-0.000002	5.5	0.0057
Saccharofermentans	0.0388	0.000001	1.2	0.0075
Veillonella	0.0397	-0.000831	86.3	0.077
Raoultella	0.0471	-0.000069	25.4	0.0094

PerStr	*P* for trend	beta	Share Rating (%)	Median relative abundance (%)
Gardnerella	0.0296	-0.000002	1.4	0.011
Acinetobacter	0.0012	-0.000001	1.7	0.0030
Succinivibrio	0.0098	-0.000174	2.5	0.12
Lachnoanaerobaculum	0.0158	-0.000001	5.7	0.0055
Pyramidobacter	0.0342	-0.000060	6.8	0.014
Methanobrevibacter	0.0208	-0.000312	14.7	0.11
Parvimonas	0.0411	-0.000015	21.9	0.0045
Solobacterium	0.0074	-0.000007	33.8	0.0071
Eubacterium	0.0038	0.000016	34.9	0.0052
Holdemanella	0.0278	0.001125	38.1	1.73
Barnesiella	0.0126	0.000191	40.1	0.15
Bilophila	0.0300	0.000126	64.0	0.20
Turicibacter	0.0252	-0.000705	65.7	0.070
Actinomyces	0.0358	-0.000060	97.0	0.052

・*P for trend* was calculated by multiple regression analysis.

・Covariates: Age＋Gender＋Smoking＋Drinking＋BMI

Share Rating indicates the proportion of subjects with a read count of one or greater.

（GrYwVege: Green & Yellow Vegetables, PerStr: Persimmon & Strawberry）
